# Associations Between APOE Genotypes and Neuropathological Outcomes in Alzheimer's Disease: Insights from the NACC Dataset

**DOI:** 10.1002/alz70855_106320

**Published:** 2025-12-24

**Authors:** Obed Okwoli Apochi, Rebecca Bernal, Yannick Joel Wadop Ngouongo, Crystal Wiedner, Biniyam A. Ayele, Muralidharan Sargurupremraj, Xueqiu Jian, Alfred Kongnyu NJAMNSHI, Nkouonlack Cyrille, José E. Cavazos, Sudha Seshadri, Alfred N. Fonteh, Bernard Fongang, Alice Nono Djotsa, Agustin Ruiz, Margaret E Flanagan, Jayandra Jung Himali, the African Initiative on Bioinformatics Online Training in Neurodegenerative Diseases (AI‐BOND)

**Affiliations:** ^1^ National Forensic Sciences University, Gandhinagar, Gujarat, India; ^2^ Glenn Biggs Institute for Alzheimer's & Neurodegenerative Diseases, UT Health San Antonio, San Antonio, TX, USA; ^3^ Glenn Biggs Institute for Neurodegenerative Diseases, University of Texas Health Science Center at San Antonio, San Antonio, TX, USA; ^4^ Glenn Biggs Institute for Alzheimer's & Neurodegenerative Diseases, University of Texas Health Science Center, San Antonio, TX, USA; ^5^ Global Brain Health Institute, San Francisco, CA, USA; ^6^ John P. Hussman Institute for Human Genomics, University of Miami Miller School of Medicine, Miami, FL, USA, Miami, FL, USA; ^7^ Addis Ababa University, Addis Ababa, Addis Ababa, Ethiopia; ^8^ UT Health San Antonio, Bordeaux, TX, USA; ^9^ University of Texas Health Science Center at San Antonio, San Antonio, TX, USA; ^10^ University of Bordeaux, Bordeaux, France; ^11^ The University of Texas Health Science Center at San Antonio, San Antonio, TX, USA; ^12^ Brain Research Africa Initiative, Yaounde, Centre, Cameroon; ^13^ The University of Yaoundé I, Yaoundé, Cameroon, Cameroon; ^14^ Faculty of Health Sciences, University of Buea, Buea, Cameroon; ^15^ Brain Research Africa Initiative, Yaoundé, Cameroon; ^16^ Neurology and Physiology UT Health San Antonio, San Antonio, TX, USA; ^17^ Glenn Biggs Institute for Alzheimer's & Neurodegenerative Diseases, University of Texas Health Science Center, San Antonio, TX, USA; ^18^ Department of Neurology, Boston University School of Medicine, Boston, MA, USA; ^19^ Framingham Heart Study, Framingham, Boston, MA, USA; ^20^ Department of Neurology, University of Texas Health Sciences Center, San Antonio, TX, USA, San Antonio, TX, USA; ^21^ University of Texas Health San Antonio, San Antonio, TX, USA; ^22^ Huntington Medical Research Institutes, Pasadena, CA, USA; ^23^ Keck School of Medicine, University of Southern California, Los Angeles, CA, USA; ^24^ Glenn Biggs Institute for Alzheimer's & Neurodegenerative Diseases, The University of Texas Health Science Center at San Antonio, San Antonio, TX, USA; ^25^ Department of Population Health Sciences, The University of Texas Health Science Center at San Antonio, San Antonio, TX, USA; ^26^ Department of Biochemistry and Structural Biology, The University of Texas Health Science Center at San Antonio, San Antonio, TX, USA; ^27^ Section of Health Services Research, Baylor College of Medicine, Houston, TX, USA; ^28^ Center for Health Services Research & Development Center for Innovation Quality, Effectiveness and Safety (IQUEST), Michael E. DeBakey VA Medical Center, Housten, TX, USA; ^29^ Joe R. and Teresa Lozano Long School of Medicine, University of Texas Health Science Center, San Antonio, TX, USA; ^30^ Research Center and Memory Clinic, Ace Alzheimer Center Barcelona, Universidad Internacional de Catalunya, Barcelona, Barcelona, Spain; ^31^ Biomedical Research Networking Centre in Neurodegenerative Diseases (CIBERNED), National Institute of Health Carlos III, Madrid, Madrid, Spain; ^32^ Ace Alzheimer Center Barcelona – International University of Catalunya (UIC), Barcelona, Spain; ^33^ Department of Microbiology, Immunology and Molecular Genetics, University of Texas Health Science Center, San Antonio, TX, USA; ^34^ CIBERNED, Network Center for Biomedical Research in Neurodegenerative Diseases, National Institute of Health Carlos III, Madrid, Spain; ^35^ Glenn Biggs Institute for Alzheimer's and Neurodegenerative Diseases, UT Health San Antonio, San Antonio, TX, USA; ^36^ Pathology, San Antonio, TX, USA; ^37^ Glenn Biggs Institute for Alzheimer's & Neurodegenerative Diseases, San Antonio, TX, USA; ^38^ The Framingham Heart Study, Framingham, MA, USA; ^39^ Boston University School of Public Health, Boston, MA, USA; ^40^ Graduate School of Biomedical Sciences, University of Texas Health Science Center at San Antonio, San Antonio, TX, USA

## Abstract

**Background:**

The *APOE* genotype, particularly *APOE*‐ε4, increases AD risk and influences neuropathology. However, its role in disease progression remains complex, varying with individual factors like age and sex, requiring further investigation. Our objective is to assess the relationship between *APOE* genotype and neuropathological outcomes and explore modifying effect of TDP‐43 proteinopathy.

**Methods:**

This study used the NACC dataset with 7,117 participants (mean age 79 ± 11.6 years), 47.6% female, 94.6% White, and 30.7% post‐master educated. Participants were classified by *APOE*‐ε2 carriers (ε*22* or ε*32)*, ε3 homozygotes (ε*33)*, and *APOE*‐ε4 carriers (ε*24*, ε*43* or ε*44)*. Neuropathological outcomes including Thal Phase, Braak Stage, neuritic plaques, diffuse plaques, and cerebral amyloid angiopathy, were examined. Logistic regression models adjusted for age, sex, self‐reported ancestry, and education were used. Additionally, to assess the modifying effect of TDP‐43 proteinopathy interaction analyses were performed. Statistical significance was set at α=0.05.

**Results:**

Compared to ε3 homozygotes, *APOE*‐ε4 carriers had significantly higher odds of Thal Phase (OR[95%CI], p: 4.70[4.09,5.41],<0.001), Braak Stage (3.78[3.42,4.18],<0.001), neuritic plaques (3.48[3.17,3.83],<0.001), diffuse plaques (3.46[3.12,3.85],<0.001) and Cerebral amyloid angiopathy (3.49[3.18,3.84],<0.001). *APOE*‐ɛ2 carriers showed reduced odds of Thal Phase (0.51[0.42,0.63], <0.001), Braak Stage (0.56 [0.48, 0.66], <0.001), neuritic plaques (0.48 [0.41, 0.56], <0.001), diffuse plaques (0.49 [0.42, 0.57], <0.001). We did not observe any other statistically significant associations.

We observed a statistically significant interaction by TDP‐43 proteinopathy in the association between *APOE* and neuritic plaques (*p* = 0.028) but not with other pathologies. The effect of *APOE*‐ε4 was reduced in individuals with any TDP‐43 presence (3.68[2.89, 4.69], <0.001) compared to those with absence of TDP‐43 (4.17[3.36, 5.17], <0.001). Interestingly, the protective effect of *APOE*‐ε2 was enhanced in individuals with any TDP‐43 presence (0.28[0.18, 0.45], <0.001) compared to those with absence of TDP‐43 (0.63[0.45, 0.87], 0.006).

**Conclusions:**

These findings suggest that TDP‐43 proteinopathy modifies the genetic risk associated with *APOE*, attenuating the impact of *APOE*‐ε4 while amplifying the protective role of *APOE*‐ε2. *APOE*‐ɛ2 carriers appear to have a protective effect. The presence of TDP‐43 proteinopathy may further modify these associations. Future studies should explore the impact of additional genetic and environmental factors on AD neuropathology burden.

